# Automated Segmentation of Forearm Muscles: Clinical Associations With Hand Function, Muscle Volume and Intramuscular Fat

**DOI:** 10.1002/rco2.70015

**Published:** 2025-10-19

**Authors:** Joel Fundaun, Valeria Oliva, Sandrine Bédard, Evert Onno Wesselink, Benjamin P. Lynn, Anoosha Pai S., Dario Pfyffer, Merve Kaptan, Nazrawit Berhe, John Ratliff, Serena S. Hu, Zachary A. Smith, Trevor J. Hastie, Sean Mackey, Marnee J. McKay, James M. Elliott, Scott L. Delp, Akshay S. Chaudhari, Christine S. W. Law, Andrew C. Smith, Kenneth A. Weber

**Affiliations:** ^1^ Division of Pain Medicine, Department of Anesthesiology, Perioperative and Pain Medicine Stanford University School of Medicine Stanford California USA; ^2^ Center for Behavioral Sciences and Mental Health Italian National Health Institute Rome Italy; ^3^ NeuroPoly Lab, Institute of Biomedical Engineering Polytechnique Montréal Montréal Québec Canada; ^4^ Department of Bioengineering Stanford University School of Medicine Stanford California USA; ^5^ Department of Neurosurgery Stanford University School of Medicine Stanford California USA; ^6^ Department of Orthopaedic Surgery Stanford University School of Medicine Stanford California USA; ^7^ Department of Neurosurgery University of Oklahoma Health Sciences Center Oklahoma City Oklahoma USA; ^8^ Department of Statistics Stanford University Stanford California USA; ^9^ The Faculty of Medicine and Health The University of Sydney Camperdown New South Whales Australia; ^10^ Northern Sydney Local Health District The Kolling Institute St. Leonards New South Whales Australia; ^11^ Department of Bioengineering and Mechanical Engineering Stanford University Stanford California USA; ^12^ Department of Radiology Stanford University School of Medicine Stanford California USA; ^13^ Physical Therapy Program, Department of Physical Medicine and Rehabilitation, School of Medicine University of Colorado Aurora Colorado USA

**Keywords:** computer‐assisted, dexterity, forearm, grip strength, image processing, magnetic resonance imaging

## Abstract

**Background:**

Hand function is critical for daily activities and declines early in many diseases, conditions or disorders affecting the musculoskeletal and neurologic systems. Muscle health markers derived from clinically available magnetic resonance imaging (MRI) scans are strongly associated with functional capacity, may enhance clinical assessment and inform management options. However, traditional muscle MRI assessments require time‐intensive manual segmentations. Here, we aim to develop and test a computer‐vision model for automated forearm muscle segmentation and investigate associations between MRI‐derived muscle markers and age, sex, BMI, functional grip strength and dexterity measures.

**Methods:**

We recruited 42 healthy, right‐handed adults (54.8% female, median age 37.3 years, median BMI: 23.0). Grip strength and dexterity were measured using the NIH Toolbox motor battery. Dixon fat‐water MRI of the right forearm was acquired at 3.0 T, and forearm flexor and extensor muscle compartments were manually segmented for model training. A 2D U‐Net convolutional neural network model was trained and tested for segmentation of the forearm flexors and extensors for the assessment of muscle volume and intramuscular fat. Testing accuracy and reliability were assessed using Sørensen–Dice indices, intraclass correlation coefficients (ICCs) and Bland–Altman analyses. Associations between the MRI‐derived muscle markers, demographic factors, muscle metrics and hand function were evaluated using partial correlations and regression models.

**Results:**

The segmentation model showed high test accuracy, achieving mean Sørensen–Dice indices of 0.89 (flexors) and 0.85 (extensors) and ICCs of 0.75–0.99 for muscle volume and intramuscular fat. Muscle volume was positively correlated with BMI (*p* < 0.001) but not age (*p* > 0.249). Males had larger muscle volumes than females (*p* < 0.001), with no sex differences in intramuscular fat (*p* > 0.141), and no association between intramuscular fat and grip strength or dexterity (*p* > 0.350). We observed strong positive correlations between grip strength and both flexor (*p* = 0.004) and extensor (*p* = 0.001) muscle volumes, while dexterity showed no significant associations.

**Conclusions:**

Our findings highlight the accuracy and reliability of automated forearm muscle segmentation using computer vision. BMI emerged as a key determinant of muscle volume, independent of age. The strong association between muscle volume and grip strength demonstrates the clinical relevance of these metrics, suggesting potential applications in therapeutic planning for conditions impairing hand function. Sex‐based differences in muscle volume underscore the importance of tailored assessments. Computer vision models integrated with Dixon fat‐water MRI enable efficient, accurate evaluation of forearm muscle health. Future research should explore these metrics in clinical populations and their utility in tracking functional outcomes.

## 1 Introduction

Hand function is critical for daily life and involves a complex interplay between the neural and musculoskeletal systems. Impaired hand function is common in many musculoskeletal, neurological and neuromuscular conditions and can be a diagnostic and prognostic indicator of disease [1, 2]. Limitations in hand function, including reduced grip strength and loss of dexterity, impact quality of life and functional independence. For example, altered grip strength has been associated with numerous health outcomes, including cognitive decline, age‐related disability, falls and mortality [3, 4, 5]. Numerous neuromuscular diseases result in altered hand dexterity, including Friedreich ataxia [6], amyotrophic lateral sclerosis [7], sensorimotor neuropathies [8] and muscular dystrophies [9]. The corresponding deficits in hand function may also impact quality of life, limit physical independence and impair communication [10].

Although typical hand function includes interactions between the muscular and nervous systems, forearm muscle health and activation are essential for adequate hand function [11]. While grip strength and dexterity are commonly used in clinical settings and represent accessible outcome measures, they do not provide insights into individuals' underlying muscle health. Previous evidence using ultrasound imaging suggests that forearm muscle thickness is correlated with grip strength in healthy older men and women [12]. However, magnetic resonance imaging (MRI) is considered the gold standard for volumetric assessment [13]. MRI‐derived muscle metrics of the forearm, including muscle volume and intramuscular fat, have previously been correlated with strength and functional capacity [14], disease duration [15] and progression [16]. Extracting and quantifying MRI muscle health metrics typically requires manually segmenting individual muscles, which is labor‐intensive, error‐prone and impractical for large‐scale research or clinical use. Implementing automated segmentation processes in clinical care may improve the understanding of pathology, prognosis and management of numerous neurological and musculoskeletal conditions.

In this study, we aimed to (1) assess the automated forearm segmentation model performance, (2) quantify forearm muscle volume and intramuscular fat using MRI and (3) evaluate the relationships between these MRI‐derived muscle markers and demographic and clinical factors such as age, sex, body mass index (BMI), grip strength and hand dexterity. Here, we provide a novel automated approach for forearm muscle segmentation and study the associations between MRI‐derived forearm muscle health markers and participants' demographics and measures of hand function.

## 2 Methods

### 2.1 Participants

We recruited 42 healthy community volunteers aged 18–70 years from Palo Alto and surrounding San Francisco Bay Area communities, CA, USA. Stanford University's Institutional Review Board (Protocol #IRB‐68782) approved the study, and each participant provided written informed consent. Inclusion criteria included being right‐handed and fluent in English. Exclusion criteria included contraindications to MRI, pregnancy or thought to be pregnant in the absence of contraception since their last normal menstrual period, history of neurological or neurodegenerative conditions, cancer, major physical trauma (e.g., broken bones, traumatic brain injury), a history of back or neck surgery, a history of substance use disorder, psychiatric or mental health conditions or any painful conditions. Participant demographic details included age, sex, height, weight and dominant handedness.

### 2.2 Hand Function Metrics

Right‐hand strength and function were measured using the NIH Toolbox motor battery [17]. Grip strength was measured with a grip dynamometer (Jamar plus dynamometer, Performance Health Supply Inc., Cedarburg, WI, USA). Participants were seated with feet flat on the floor and their elbows bent at 90°. After a practice trial to familiarize themselves with the device, participants performed a single test trial, squeezing the dynamometer handle as hard as possible for 3 s. We recorded the maximum force during the test, which we used for analysis. Right‐hand dexterity was evaluated using the 9‐Hole Pegboard test (Jamar 9 Hole Peg Test kit, Performance Health Supply Inc., Cedarburg, WI, USA). Participants completed one practice trial followed by a single test trial, per NIH Toolbox recommendations. Seated with the pegboard positioned in front of them and pegs aligned closest to their right hand, participants placed the pegs into the holes one at a time and then removed them as quickly as possible. The total time taken to complete the test trial was recorded for analysis. We transformed uncorrected hand strength and dexterity values into corrected *T* scores adjusted for age, sex, education, race and ethnicity according to NIH Toolbox recommendations (mean scores of 50 and standard deviations of 10) [18].

### 2.3 Forearm Image Acquisition

Right forearm fat and water separated axial MRI was performed on a 3.0‐T General Electric SIGNA Premier scanner using a 6‐point Dixon sequence (IDEAL, TR/TE = 14/7 ms, number of echoes = 6, flip angle = 5°, averages = 2, acquisition matrix = 128 × 128, field‐of‐view = 30 cm, slice thickness = 3 mm, scan time = 4 min 37 s) and a 20‐channel AIR flex coil. Participants were lying on the right side with the arm flexed, elbow extended and forearm above the head and positioned in the centre of the scanner bed.

### 2.4 Forearm Segmentation

The extensor and flexor compartments of the forearm, radius and ulna were manually segmented using 3D Slicer using the axial fat, water, in‐phase, out‐of‐phase, fat fraction and R_2_* images (Version 5.6.1) [19] by a single rater trained in forearm anatomy. The distal and proximal ends spanned approximately from the humeroulnar to the radio‐carpal joints to the proximal border of pronator quadratus. Pronator quadratus was not segmented. Flexor carpi ulnaris, palmaris longus, flexor carpi radialis, pronator teres, flexor digitorum superficialis, flexor digitorum profundus and flexor pollicis longus were included in the flexor compartment, and extensor carpi radialis longus, extensor carpi radialis brevis, extensor carpi ulnaris, extensor digitorum, extensor digiti minimi, abductor pollicis, extensor pollicis longus, extensor pollicis brevis, and extensor indicis were included in the extensor compartment. The forearm imaging had insufficient resolution and contrast to confidently identify smaller individual muscle borders, and the muscles were grouped into the previously described flexor and extensor compartments.

### 2.5 Computer‐Vision Model

**FIGURE 1:**
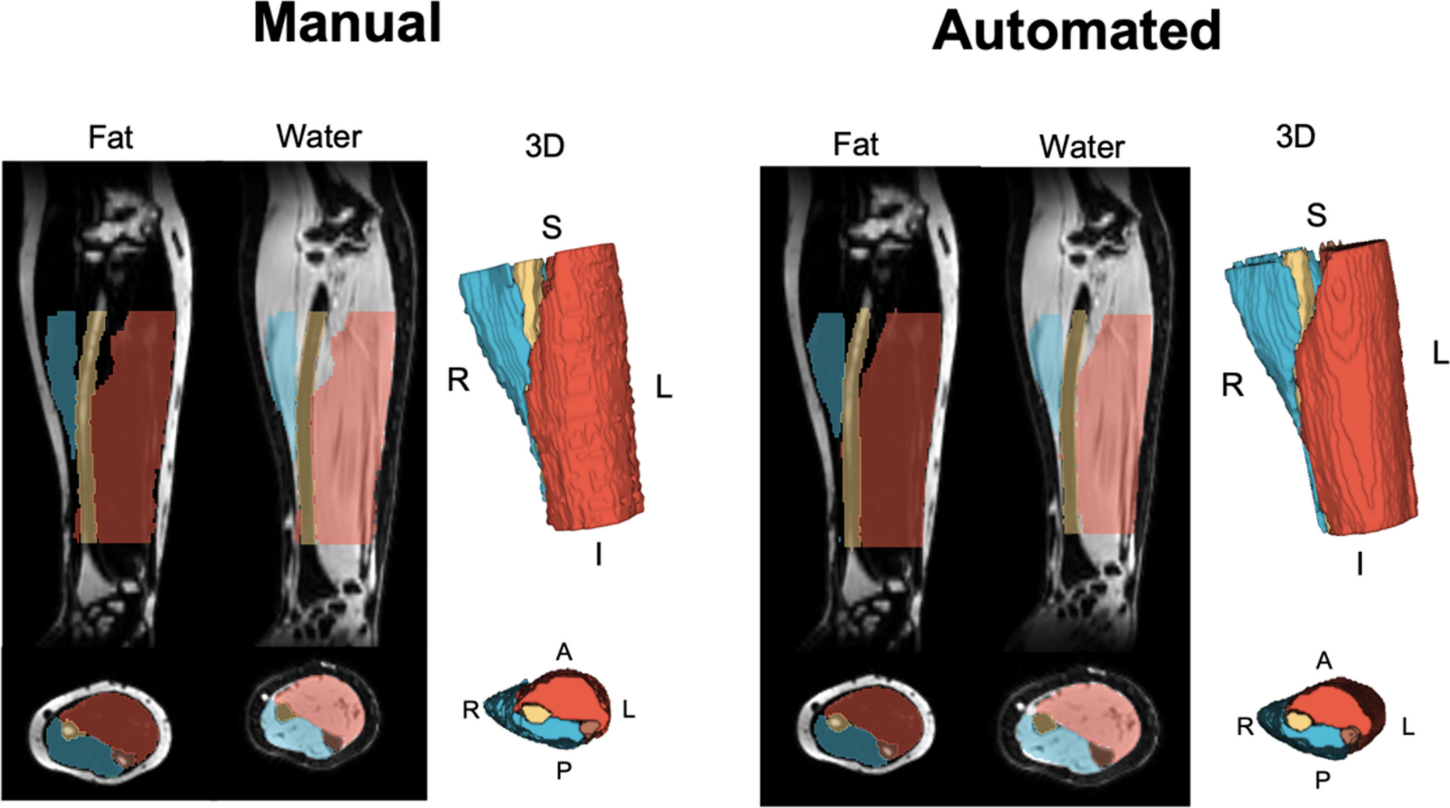
Comparison of manual and automated forearm segmentations. Axial and sagittal forearm images with overlaid manual and automated segmentations. Segmentations are displayed on fat and water images and include 3D renderings. Segmented forearm structures include the flexor muscles (red), extensor muscles (blue), radius (yellow) and ulna (brown).

A 2D U‐Net convolutional neural network computer‐vision model was trained and tested for multiclass segmentation using the manual segmentations from the images described above, using the MONAI Python package [20, 21] (Figure 1). The dataset was split into Training (66.7% of participants) and Testing (33.3%) sets, proportionally matched by sex (*p* = 0.67), age (*p* = 0.60), and BMI (*p* = 0.37). Image preprocessing included intensity normalization through *z*‐score normalization. Image augmentation techniques included random affine transformations with 10% probability (translation: 10 voxels in *x*, *y*, *z*; rotation: 16° in *x*, *y*, *z*; scaling: 15%; histogram shifting: number of control points; contrast adjustment: gamma 0.2–2; bias field distortion: degree = 3, coefficient range −1 to 1; Gaussian noise: mean = 0.0, SD = 0.1; and Gibbs noise simulation: alpha 0.3–0.9). We used a U‐Net model [22] with a spatial window size of 128 × 128, a batch size of 2, a spatial window batch size of 10, LeakyReLU activation (negative slope 0.01), instance normalization, two residual units per level, and a stride of 2 at each down sampling step. The model was trained for 168 000 iterations, corresponding to 1000 epochs. The loss function was soft Dice‐Cross Entropy (equally balanced) with softmax activation and one‐hot encoding. Stochastic Gradient Descent with momentum (0.99), Nesterov acceleration and weight decay (1 × 10^−4^) were used. A polynomial learning rate scheduler (power = 0.9) was applied, with an initial learning rate of 0.01. Mixed precision (AMP) was enabled for computational efficiency. Sliding window inference was performed with a window size of 128 × 128, an overlap ratio of 0.25, and Gaussian‐weighted blending to reduce edge artefacts. Model training and testing were performed using a NVIDIA RTX 3090 24‐GB graphical processing unit (GPU, NVIDIA, Santa Clara, CA).

### 2.6 Statistical Analysis

Muscle volume and intramuscular fat estimated from the U‐Net were compared with manual muscle segmentations. Segmentation masks were used to delineate specific forearm compartments, including the radius, ulna, extensor compartment and flexor compartment. Binary masks corresponding to each compartment were applied to isolate relevant voxels from the fat and water images. Mean fat and water signals were then calculated from the voxels within the segmentation masks. Intramuscular fat was quantified as the percentage of the mean fat signal relative to the total mean signal (fat + water) within each muscle compartment obtained from Dixon‐based scans.

Normality was assessed via the Shapiro–Wilk test and visual inspection. We assessed model voxel classification accuracy by comparing ground manual to automated segmentation methods using multiple measures, including Sørensen–Dice index, Jaccard index, conformity coefficient, true positive rate, true negative rate, positive predictive value and volume ratio. The reliability of muscle volume and intramuscular fat estimates in the testing set (*n* = 14) was assessed using two‐way random effects intraclass correlation coefficients (ICC2,1) with 95% confidence intervals and Bland–Altman plots, accounting for differences across subjects. Associations between forearm muscle volume, intramuscular fat, age and BMI were assessed using partial correlation, adjusting for sex and either BMI or age as applicable. Ordinary Least Squares regression was first used to obtain residual values by regressing muscle volume and intramuscular fat on sex and the alternate variable (BMI or age). Pearson's correlation was then computed between the residual values to estimate partial correlations. ANCOVA assessed sex effects on muscle volume and intramuscular fat, controlling for age and BMI. Multiple linear regression examined relationships between grip strength, dexterity, muscle volume and intramuscular fat, controlling for age, BMI and sex. All image and statistical analyses were completed using Python (version 3.13.0). *p* < 0.05 indicates statistical significance.

## 3 Results

### 3.1 Participant Characteristics

Forty‐two healthy participants were included (54.8% female, median age 37.3 years [IQR 13.0], median BMI: 23.0 [IQR 4.8], Table 1). Mean corrected grip strength and dexterity *T* scores were 45.2 (SD 8.3) and 56.4 (SD 11.2), respectively.

**TABLE 1:** Participant characteristics.

	Total cohort (*n* = 42)	Training (*n* = 28)	Testing (*n* = 14)
Age, median (IQR)	37.3 (13.0)	36.5 (12.6)	38.9 (14.2)
Sex, % female (*n*)	54.8% (23/42)	57.1% (16/28)	50% (7/14)
BMI, median (IQR)	23.0 (4.8)	22.7 (4.9)	23.2 (3.3)
Grip strength corrected *T* score, mean (SD)	45.2 (8.3)	44.4 (8.8)	47.0 (7.1)
Dexterity corrected *T* score, mean (SD)	56.4 (11.2)	57.8 (12.0)	53.3 (9.2)

Abbreviations: IQR = interquartile range, SD = standard deviation.

### 3.2 Segmentation Model Performance

Figure 1 illustrates the manual and automated segmentations from a representative image from the testing set.

The automated model achieved high accuracy with manual segmentations (Tables 2 and 3, Figure S1). Mean Sørensen–Dice index values were 0.85 (SD 0.1) for the extensor compartment, 0.89 (SD 0.1) for the flexor compartment and 0.82 (SD 0.01) for both the radius and the ulna (Table 2). Mean Jaccard Index values were 0.76 (SD 0.01) for the extensor compartment, 0.82 (SD 0.00) for the flexor compartment and 0.71 (SD 0.01) for both the radius and the ulna. The conformity coefficient was similar for the extensor compartment (0.68 [SD 0.01]) and the flexor compartment (0.78 [SD 0.01]), 0.58 (SD 0.01) for the radius and 0.59 (SD 0.01) for the ulna. Reports of all automated model segmentation accuracy measures are listed in Table 2.

**TABLE 2:** Automated model segmentation accuracy.

Structure	Dice	JI	CC	TPR	TNR	PPV	VR
Radius	0.82 (0.01)	0.71 (0.01)	0.58 (0.01)	0.80 (0.01)	1.00 (0.00)	0.86 (0.01)	0.94 (0.01)
Ulna	0.82 (0.01)	0.71 (0.01)	0.59 (0.01)	0.82 (0.01)	1.00 (0.00)	0.84 (0.01)	0.98 (0.01)
Extensor compartment	0.85 (0.01)	0.76 (0.01)	0.68 (0.01)	0.85 (0.01)	1.00 (0.00)	0.88 (0.01)	0.98 (0.01)
Flexor compartment	0.89 (0.01)	0.82 (0.00)	0.78 (0.01)	0.88 (0.01)	1.00 (0.00)	0.93 (0.00)	0.95 (0.01)

*Note:* The automated model accuracy was compared with manual segmentations of the Testing dataset (*n* = 14) using the Sørensen–Dice index (Dice), Jaccard index (JI), conformity coefficient (CC), true positive rate (TPR), true negative rate (TNR), positive predictive value (PPV) and volume ratio (VR). Metrics shown = average (standard error).

**TABLE 3:** Muscle volume and IMF reliability.

Measurement	ICC	95% lower CI	95% upper CI	*p* value
**Volume (mL)**				
Radius	0.94	0.80	0.98	< 0.001
Ulna	0.97	0.90	0.99	< 0.001
Extensor compartment	0.99	0.97	1.00	< 0.001
Flexor compartment	0.99	0.98	1.00	< 0.001
**Fat (%)**				
Extensor compartment	0.75	0.27	0.92	0.008
Flexor compartment	0.96	0.87	0.99	< 0.001

*Note:* Automated model reliability was assessed on the training and testing datasets (*n* = 42) using intraclass correlation coefficients (ICC_2,1_).

Abbreviations: CI = confidence interval, ICC = intraclass correlation coefficient.

The automated model demonstrated excellent reliability for the extensor (ICC_2,1_ = 0.99, 95% CI: 0.97–1.0, *p* < 0.001) and the flexor compartments (ICC_2,1_ = 0.99, 95% CI: 0.98–1, *p* < 0.001), as well as for the radius (ICC_2,1_ = 0.94, 95% CI: 0.80–0.98, *p* < 0.001) and ulna (ICC_2,1_ = 0.97, 95% CI: 0.90–0.99, *p* < 0.001) (Table 3). The reliability of intramuscular fat for the extensor compartment was 0.75 (95% CI: 0.27–0.92, *p* = 0.008) and 0.96 (95% CI: 0.87–0.99, *p* < 0.001) for the flexor compartment.

### 3.3 Age, BMI and Sex

**FIGURE 2:**
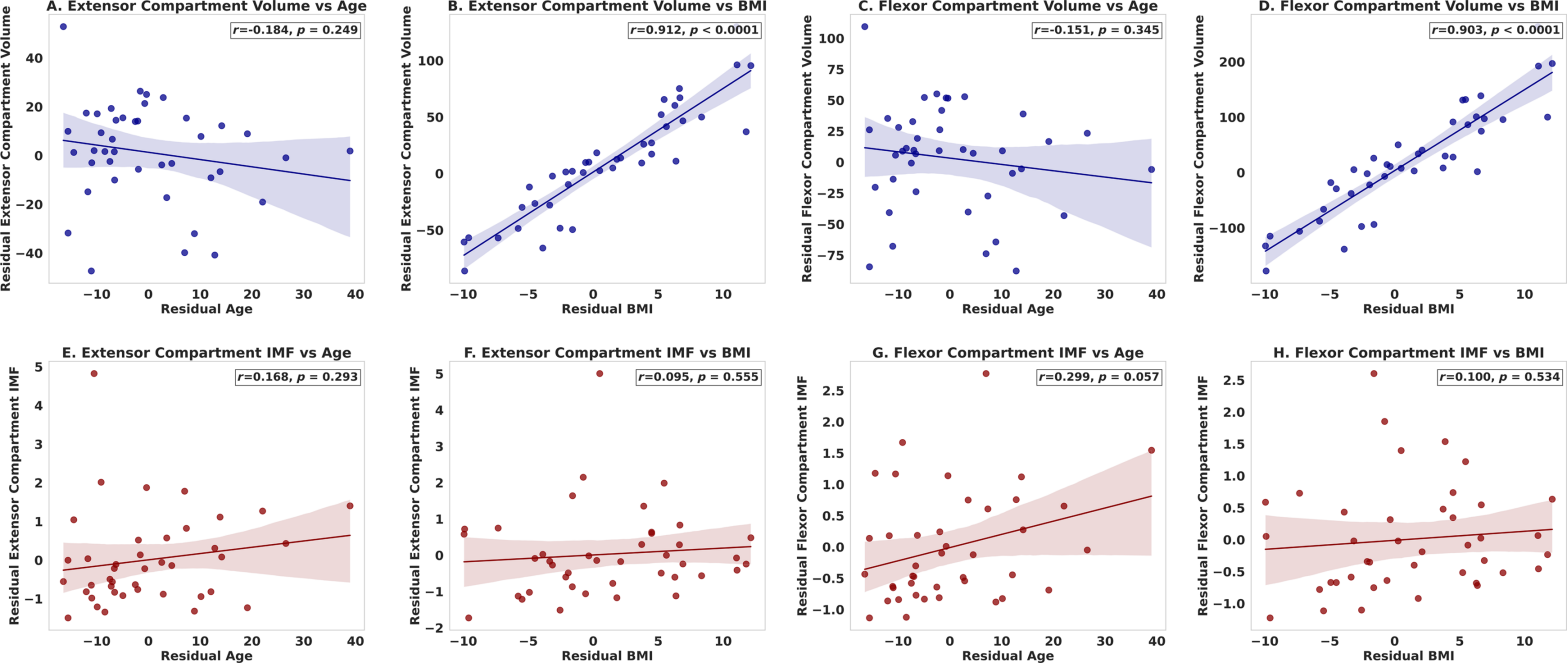
Correlation of automated forearm muscle volume and intramuscular fat with age and BMI. Partial correlation analyses (Pearson's *r*) were conducted to assess the relationships between forearm muscle volumes, intramuscular fat, age and BMI across 42 participants (23 females, 19 males). Panels A–D display residual muscle volume for the extensor and flexor compartments plotted against residual age and BMI, adjusting for sex and the alternate variable (BMI/age) as applicable. Panels E–H depict residual intramuscular fat in the flexor and extensor compartments relative to residual age and BMI, controlling for sex and the alternate variable (BMI/age). BMI = body mass index.

**FIGURE 3:**
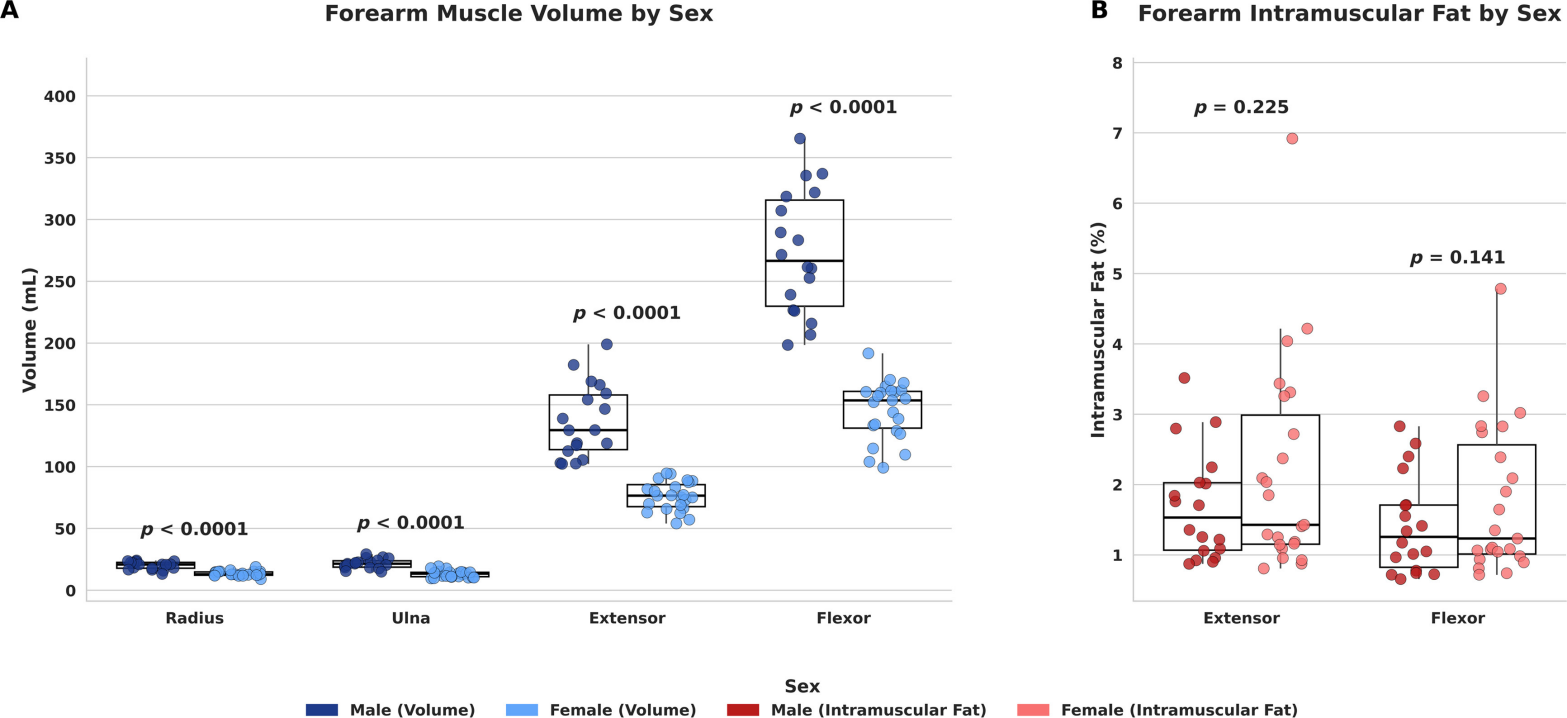
Automated forearm volumes and intramuscular fat sex differences. Sex differences in automated forearm (A) volume and (B) intramuscular fat were evaluated using ANCOVA, controlling for age and BMI, in 42 participants (23 females, 19 males).

Forearm extensor (*r* = 0.912, *p* < 0.0001) and flexor muscle volumes (*r* = 0.903, p < 0.0001) were strongly positively correlated with BMI but not age (extensor volume: *r* = −0.184, *p* = 0.249, flexor volume: *r* = −0.151, *p* = 0.345, Figure 2A–D). Intramuscular fat of the forearm extensors was not correlated with age (*r* = 0.168, *p* = 0.293) or BMI (*r* = 0.095, *p* = 0.555). Intramuscular fat of the forearm flexors was also not correlated with age (*r* = 0.299, *p* = 0.057) or BMI (*r* = 0.100, *p* = 0.534, Figure 2E–H). Males exhibited larger forearm extensor and flexor muscle volumes than females (*p* < 0.001, Figure 3A). The volume of the radius and ulna was also larger in males compared with females (*p* < 0.001). There were no sex differences in intramuscular fat comparing males and females in the forearm extensor (*p* = 0.225) and flexor muscles (*p* = 0.141, Figure 3B).

### 3.4 Grip Strength and Dexterity

Linear regression analysis demonstrated that the flexor (coefficient = 0.110, *p* = 0.001) and extensor compartment muscle volumes (coefficient = 0.210, *p* = 0.004) were associated with computed grip strength *T* scores (Table 4). However, muscle volumes for the flexor (coefficient = 0.063, *p* = 0.194) and extensor compartments (coefficient = 0.122, *p* = 0.220) were not associated with computed dexterity *T* scores. Neither grip strength nor dexterity *T* scores were associated with intramuscular fat (*p* > 0.350).

**TABLE 4:** Association of automated forearm muscle volume and fat with corrected grip strength and dexterity scores.

Grip strength (*T* score)								
Group	Volume	*p*	*F*	Adj *R* ^2^	IMF	*p*	*F*	Adj *R* ^2^
Flexor compartment	0.110 (0.03)	**0.001**	322.9	0.97	0.109 (0.11)	0.951	232.2	0.96
Extensor compartment	0.210 (0.07)	**0.004**	306.8	0.97	1.202 (1.643)	0.470	236.2	0.96

Finger dexterity (*T* score)								
Group	Volume	*p*	*F*	Adj *R* ^2^	IMF	*p*	*F*	Adj *R* ^2^
Flexor compartment	0.063 (0.05)	0.194	222.0	0.96	−2.162 (2.280)	0.350	216.1	0.96
Extensor compartment	0.122 (0.10)	0.220	220.6	0.96	−1.521 (2.155)	0.485	213.4	0.96

*Note:* Correct grip strength and dexterity *T* scores were adjusted for age, sex, education, race, and ethnicity. Standardized coefficients (standard error). *p* = *p* values of coefficient estimate, IMF = intramuscular fat, BMI = body mass index, Adj *R* ^2^ = variance explained including age, sex and BMI. Statistical significance indicated by bolded text.

Similarly, muscle volumes for the flexor (coefficient = 0.345, *p* < 0.001) and extensor compartments (coefficient = 0.650, *p* < 0.001) were associated with uncorrected grip strength scores (Table S1). Muscle volumes for the flexor (coefficient = 0.039, *p* = 0.001) and extensor compartments (coefficient = 0.077, *p* = 0.002) were also associated with uncorrected dexterity scores (Table S2). Grip strength and dexterity uncorrected scores were also not associated with intramuscular fat (*p* > 0.410).

## 4 Discussion

Accurately assessing and quantifying muscle health is critical for evaluating and tracking health and disease. Manual segmentation is inefficient and error‐prone. Automated, scalable methods for muscle segmentation are critical to assess muscle health in large‐scale research applications and to translate these markers to clinical practice. We developed a computer‐vision–based segmentation model to automatically segment the forearm flexor and extensor muscles using fat‐water imaging data from healthy participants. Using the automated measures, we show that forearm muscle volumes are strongly associated with BMI but do not appear to be associated with age. We also demonstrate that forearm flexor and extensor muscle volumes are significantly associated with grip strength.

Our findings reveal significant associations between forearm muscle volumes and BMI, independent of age. This association is not surprising, as BMI reflects overall body mass, and individuals with greater body mass typically exhibit larger absolute muscle volumes. These findings also align with previous research indicating the role of body composition and body size in skeletal muscle health [23, 24]. To note, BMI is used clinically as a crude and limited measure of healthy weight; however, BMI only considers body mass and height and does not differentiate between fat and muscle mass. Interestingly, age was not significantly associated with forearm muscle volumes in our cohort of healthy individuals; however, the sign of the associations was negative for the forearm extensors and flexors, suggesting age‐related atrophy. The lack of significant associations between forearm muscle volume and age may be due to the younger age range of our sample (median 37.3 years [IQR 13.0]). A larger sample with greater variance in age may be needed to better characterize age‐related atrophy of the forearm muscles. Similarly, Vidt et al. reported no significant differences in forearm flexor and extensor muscle volumes when comparing older and younger adults [25], further reinforcing that forearm muscles may be less affected by age‐related atrophy. Our recent work evaluating muscle metrics in the leg also did not identify an association between leg muscle volume and age [26]. While age is a well‐established factor in muscle health for other regions, such as the trunk [27] and thigh muscles [28, 29], our results suggest potential regional variations in muscle aging processes. This finding underscores the importance of future research evaluating potential mechanisms underlying these regional differences and their implications for muscle health across the lifespan.

As expected, we observed sex differences in both forearm flexor and extensor muscle volumes, with females having smaller muscle volumes than males, indicating the need for tailored approaches in muscle health assessments. Similarly, a study using ultrasound imaging suggested greater cross‐sectional areas of the flexor carpi radialis and flexor digitorum superficialis muscles in males compared with females [30]. Prior research in other body regions has also reported sex differences in muscle volume and cross‐sectional area, including the thigh [31, 32], cervical [33] and lumbar paraspinal muscles [14, 26, 34]. Although we did not observe significant differences in intramuscular fat between sexes, our data show trends of females having higher intramuscular fat in the forearm flexors and extensors, as previously identified [14, 33]. For both sexes, we identified greater muscle volumes in the wrist flexors than extensors, consistent with previous MRI‐derived volumetrics of individually labelled forearm muscles, which indicated that flexors are approximately twice the size of extensors [35].

A unique aspect of our study is the integration of hand function and automated forearm muscle segmentation metrics. Hand function, including grip strength and hand dexterity, is critical for daily activities and becomes significantly impaired and associated with numerous health outcomes and mortality [3, 4, 5]. While grip strength serves as a valuable and accessible marker, it lacks specificity in identifying the underlying muscular or neuromuscular contributors to function. Our findings demonstrate strong associations between forearm muscle volumes and grip strength, highlighting the potential value of quantitative MRI measures to elucidate underlying morphometric muscular changes associated with grip strength.

We further investigated the relationship between forearm muscle metrics and hand function by assessing associations with hand dexterity, a measure particularly sensitive to neuromuscular impairments. Neurological conditions can often affect fine motor skills before grip strength [36], and understanding the contributions of specific forearm muscle compartments may inform early diagnosis, provide prognostic value and inform targeted therapies. Our findings using corrected dexterity *T* scores, which we adjusted for age, sex, education, race and ethnicity based on normative values from a large, representative sample of healthy individuals in the United States [17], did not identify associations between forearm muscle volume or intramuscular fat and dexterity in healthy adults. In contrast, our analyses with uncorrected dexterity scores demonstrated positive associations with both flexor and extensor compartment volumes. However, this relationship was likely influenced by sex, which showed a significant positive association with uncorrected dexterity scores in our data, while age and BMI were not associated with dexterity. However, these relationships could differ in pathological states that include alterations in neuromuscular control. As such, quantitative imaging including automated muscle segmentation could provide important insights related to disease mechanisms or prognosis. Future research in diverse populations, including those with neuromuscular disorders or aging‐related impairments, is warranted to clarify the role of forearm muscle morphometry in dexterity and fine motor function.

Previous studies have similarly demonstrated correlations between MRI‐derived muscle markers and functional outcomes. For example, muscle volume has been associated with knee flexor and extensor strength [29, 37], grip strength [29], cross‐sectional area of the erector spinae and extensor strength [38]. Our findings of a positive association between forearm flexor and extensor muscle volume and grip strength add to this evidence by highlighting the relevance of forearm muscle imaging. For example, degenerative cervical myelopathy, which is the most common form of spinal cord injury in adults [39] and can present with progressively worsening neurological status, including diminished hand function, is a consideration for surgical intervention [40]. We are currently applying the automated pipelines described here to evaluate MRI‐derived metrics of forearm muscle health to predict surgical outcomes in individuals with degenerative cervical myelopathy.

Here, we build on our previous work in the automated assessment of muscle health from MRI [14, 26, 41, 42, 43], presenting an accurate and reliable approach for segmenting forearm muscles. The application of computer vision‐based segmentation models to medical imaging has grown rapidly and is being incorporated into clinical settings [44, 45], facilitating the identification and tracking of markers across diverse anatomical regions. We have developed the MuscleMap Toolbox [41], an open‐source segmentation resource for whole‐body quantitative muscle MRI. The MuscleMap Toolbox has successfully been applied to multiple regions, including the cervical [43] and lumbar spine [14], shoulder [46], gluteal muscles [42] and legs [26] underscoring the versatility of these advanced imaging techniques. This study extends the MuscleMap Toolbox by developing a computer‐vision model to automatically segment the forearm flexor and extensor muscle compartments.

Automated forearm muscle segmentation has significant clinical relevance beyond research, potentially impacting patient care across various musculoskeletal and neurological conditions. MRI‐based biomarkers of muscle volume and intramuscular fat could enhance diagnostic precision, guide treatment decisions and monitor disease progression in conditions like inflammatory myositis [15, 47], Duchenne's muscular dystrophy [16], myotonic dystrophy [48] and cervical myelopathy [47]. In degenerative cervical myelopathy, automated assessment of forearm muscle health could inform surgical decision‐making and predict functional outcomes. This technology may enable earlier intervention, personalized treatment strategies and streamlined evaluation of therapeutic interventions in clinical trials. Implementing automated muscle segmentation could allow for more precise quantification of muscle health regarding patients' response to treatment, disease management and prognosis that functional measures, such as grip strength and dexterity, are unable to provide. By providing objective, reproducible measurements of muscle health, automated segmentation techniques have the potential to improve clinical assessment, enhance prognostic capabilities and lead to better patient outcomes across neuromuscular and musculoskeletal diseases.

### 4.1 Limitations

While our model accurately segments major components of the forearm, certain limitations should be acknowledged. The model does not resolve individual muscles within the flexor or extensor compartments due to the complexity and variability of these small structures. However, this limitation may be mitigated by the composite muscle segmentations, which still provide clinically relevant insights. Our sample size may have also constrained our ability to detect significant differences in clinical associations, particularly for intramuscular fat and age measurements between sexes. Our cohort was young and did not include equal numbers of individuals in each decade (median age 37). This may be one explanation for the lack of association with forearm muscle volume and age. Developing accurate and reliable models that generalize well across populations requires diverse datasets that capture the sample's variability. Open‐access, multisite imaging datasets would facilitate the creation of diverse cohorts to validate these findings and ensure they are representative of the broader population. Finally, our study focused on healthy individuals with a generally higher socioeconomic status and from a specific region. Hence, the applicability of these findings to clinical populations requires further testing and multisite validation.

## 5 Conclusions

We have developed the first accurate and reliable open‐source computer‐vision model for automated evaluation of forearm muscle volume and intramuscular fat, demonstrating significant associations with hand function. Our results suggest the need to consider the associations of age, sex and BMI when interpreting forearm muscle health measures. This automated approach enables efficient, scalable assessment of muscle health, with potential applications in both large‐scale research and clinical practice. While our study was limited to healthy individuals, future research should investigate these automated markers in diverse clinical populations, particularly those with conditions affecting hand function, such as degenerative cervical myelopathy or neuromuscular disorders. By providing objective, reproducible measurements of muscle health, this work adds novel insights into forearm muscle health metrics to the field of quantitative muscle imaging to ultimately improve clinical assessments and treatment planning across a range of musculoskeletal and neurological conditions.

## Author Contributions

**Joel Fundaun:** formal analysis (lead), investigation (equal), methodology (equal), validation (equal), visualization (lead), writing – original draft (lead), writing – review and editing (lead); **Valeria Oliva:** conceptualization (supporting), data curation (supporting), methodology (supporting), writing – review and editing (supporting); **Sandrine Bédard:** conceptualization (supporting), data curation (supporting), methodology (supporting), writing – review and editing (supporting); **Evert Onno Wesselink:** data curation (supporting), writing – review and editing (supporting); **Benjamin P. Lynn:** conceptualization (supporting), data curation (supporting), writing – review and editing (supporting); **Anoosha Pai S.:** data curation (supporting), investigation (supporting), writing – review and editing (supporting); **Dario Pfyffer:** conceptualization (supporting), data curation (supporting), methodology (supporting), writing – review and editing (supporting); **Merve Kaptan:** data curation (supporting), formal analysis (supporting), methodology (supporting), writing – review and editing (supporting); **Nazrawit Berhe:** data curation (supporting), writing – review and editing (supporting); **John Ratliff:** investigation (supporting), methodology (supporting), writing – review and editing (supporting); **Serena S. Hu:** methodology (supporting), writing – review and editing (supporting); **Zachary A. Smith:** investigation (supporting), methodology (supporting), writing – review and editing (supporting); **Trevor J. Hastie:** investigation (supporting), methodology (supporting), writing – review and editing (supporting); **Sean Mackey:** conceptualization (supporting), methodology (supporting), supervision (supporting), writing – review and editing (supporting); **Marnee J. McKay:** investigation (supporting), writing – review and editing (supporting); **James M. Elliott:** conceptualization (supporting), methodology (supporting), writing – review and editing (supporting); **Scott L. Delp:** methodology (supporting), writing – review and editing (supporting); **Akshay S. Chaudhari:** methodology (supporting), writing – review and editing (supporting); **Christine S. W. Law:** conceptualization (supporting), investigation (supporting), methodology (supporting), writing – review and editing (supporting); **Andrew C. Smith:** conceptualization (supporting), methodology (supporting), writing – review and editing (supporting); **Kenneth A. Weber:** conceptualization (lead), data curation (supporting), formal analysis (supporting), funding acquisition (lead), investigation (lead), methodology (supporting), project administration (lead), supervision (lead), writing – original draft (supporting), writing – review and editing (supporting).

## Ethics Statement

We confirm that we have read the Journal's position on issues involved in ethical publication and affirm that this report is consistent with those guidelines.

## Conflicts of Interest

The authors declare no conflicts of interest.

## Supporting information


**Figure S1:** rco270015‐sup‐0001‐supplemental.docx. **Correlation and Bland–Altman Plots Comparing Manual and Automated Segmentations for Muscle Volume and Intramuscular Fat.** Comparison of manual annotations and automated predictions for muscle volume (mL) and intramuscular fat (IMF, %) across the radius, ulna, extensor and flexor compartments. Correlation plots (left columns) evaluate the linear association between manual and automated method, with the solid black line indicating the regression fit, the dashed grey line representing perfect agreement (y = x) with Pearson correlation coefficient (r). Bland–Altman plots (right columns) with the dashed black line representing the mean difference (bias) and the grey dashed lines showing the 95% limits of agreement. Abbreviations: IMF: Intramuscular Fat.
Table S1: Linear regression results for uncorrected grip strength scores.
Table S2: Linear regression results for uncorrected Dexterity Scores.

## Data Availability

The deidentified datasets used in this study are available from the corresponding author upon reasonable request. The computer‐vision segmentation model was developed using open‐source Python packages (Pytorch and MONAI). We are making the computer‐vision model and associated scripts openly available for transparency, replication, reproduction and further research in more diverse samples. These will be made available on GitHub (https://github.com/MuscleMap).
