# Supplementary material for: Automated Segmentation of Forearm Muscles: Clinical Associations With Hand Function, Muscle Volume and Intramuscular Fat
Source: JCSM Commun. 2025 Oct 19;8(2):e70015. doi: 10.1002/rco2.70015 (PMC12817650; doi:10.1002/rco2.70015)
Supplement: Supplementary file 1 — Figure S1: rco270015‐sup‐0001‐supplemental.docx. Correlation and Bland–Altman Plots Comparing Manual and Automated Segmentations for Muscle Volume and Intramuscular Fat. Comparison of manual annotations and automated predictions for muscle volume (mL) and intramuscular fat (IMF, %) across the radius, ulna, extensor and flexor compartments. Correlation plots (left columns) evaluate the linear association between manual and automated method, with the solid black line indicating the regression fit, the dashed grey line representing perfect agreement (y = x) with Pearson correlation coefficient (r). Bland–Altman plots (right columns) with the dashed black line representing the mean difference (bias) and the grey dashed lines showing the 95% limits of agreement. Abbreviations: IMF: Intramuscular Fat. Table S1: Linear regression results for uncorrected grip strength scores. Table S2: Linear regression results for uncorrected Dexterity Scores. [file RCO2-8-e70015-s001.docx]

**Supplementary Materials**

**
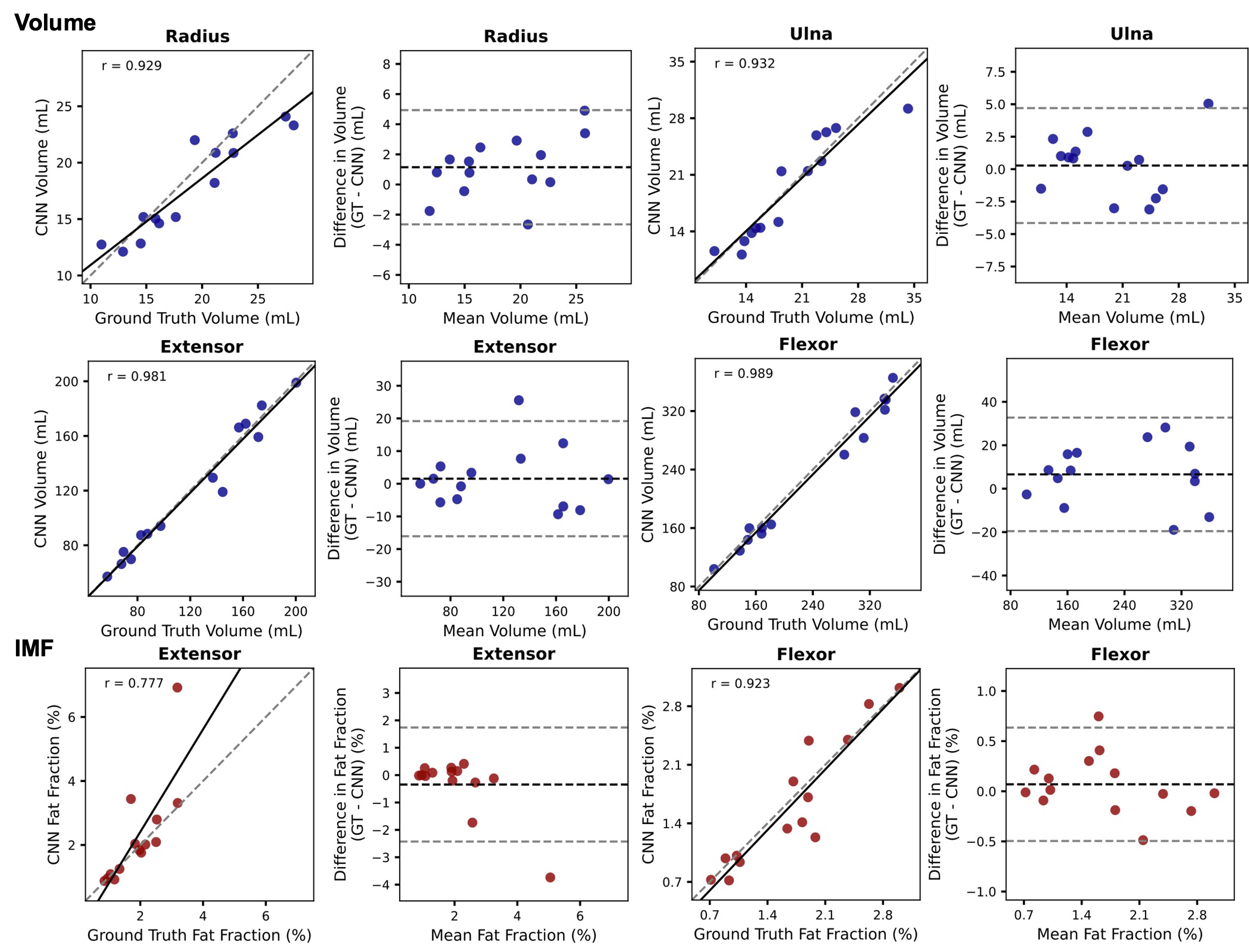
**

**Supplementary Figure 1.** **Correlation and Bland-Altman Plots Comparing Manual and Automated Segmentations for Muscle Volume and Intramuscular Fat.** Comparison of manual annotations and automated predictions for muscle volume (mL) and intramuscular fat (IMF, %) across the radius, ulna, extensor and flexor compartments. Correlation plots (left columns) evaluate the linear association between manual and automated method, with the solid black line indicating the regression fit, the dashed gray line representing perfect agreement (y = x) with Pearson correlation coefficient (r). Bland-Altman plots (right columns) with the dashed black line representing the mean difference (bias) and the gray dashed lines showing the 95% limits of agreement. Abbreviations**:** IMF: Intramuscular Fat.

**Supplementary Table 1. Linear regression results for uncorrected grip strength scores.**

|  | **Extensor Compartment Volume (mL)** | **Flexor Compartment Volume (mL)** | **Extensor Compartment Fat (%)** | **Flexor Compartment Fat (%)** |
| --- | --- | --- | --- | --- |
| Independent Variable Coefficient | 0.65 | 0.35 | 1.58 | -2.19 |
| Independent Variable Std Error | 0.13 | 0.06 | 3.76 | 4.00 |
| Independent Variable P-value | <0.001 | <0.001 | 0.68 | 0.59 |
| Age Coefficient | -0.03 | -0.05 | -0.33 | -0.25 |
| Age Std Error | 0.21 | 0.19 | 0.28 | 0.28 |
| Age P-Value | 0.89 | 0.77 | 0.24 | 0.37 |
| BMI Coefficient | 0.72 | 0.31 | 5.61 | 5.67 |
| BMI Std Error | 1.06 | 0.98 | 0.55 | 0.55 |
| BMI P-value | 0.51 | 0.75 | <0.001 | <0.001 |
| Sex Coefficient | -2.38 | 2.12 | -28.88 | -27.6 |
| Sex Std Error | 6.92 | 6.67 | 6.26 | 6.22 |
| Sex P-value | 0.73 | 0.75 | 0 | 0 |
| F-statistic | 266.4 | 313.6 | 146.8 | 147.4 |
| Adjusted R^2^ | 0.97 | 0.97 | 0.94 | 0.94 |

Uncorrected grip strength (in pounds) were not adjusted for age, sex, education, race, and ethnicity.

BMI = body mass index

Adj R^2^ = variance explained including age, sex, and BMI

**Supplementary Table 2. Linear regression results for uncorrected Dexterity Scores.**

|  | **Extensor Compartment Volume (mL)** | **Flexor Compartment Volume (mL)** | **Extensor Compartment Fat (%)** | **Flexor Compartment Fat (%)** |
| --- | --- | --- | --- | --- |
| Independent Variable Coefficient | 0.08 | 0.04 | 0.48 | 0.09 |
| Independent Variable Std Error | 0.02 | 0.01 | 0.57 | 0.61 |
| Independent Variable P-value | <0.01 | <0.01 | 0.41 | 0.89 |
| Age Coefficient | -0.01 | -0.02 | -0.05 | -0.05 |
| Age Std Error | 0.04 | 0.04 | 0.04 | 0.04 |
| Age P-Value | 0.75 | 0.65 | 0.21 | 0.3 |
| BMI Coefficient | 0.07 | 0.05 | 0.65 | 0.65 |
| BMI Std Error | 0.19 | 0.18 | 0.08 | 0.08 |
| BMI P-value | 0.69 | 0.77 | <0.001 | <0.001 |
| Sex Coefficient | 5.34 | 5.72 | 2.1 | 2.25 |
| Sex Std Error | 1.21 | 1.24 | 0.95 | 0.95 |
| Sex P-value | <0.01 | <0.01 | 0.03 | 0.02 |
| F-statistic | 390.74 | 403.62 | 291.99 | 285.7 |
| Adjusted R^2^ | 0.98 | 0.98 | 0.97 | 0.97 |

Uncorrected dexterity scores (in seconds) were not adjusted for age, sex, education, race, and ethnicity.

BMI = body mass index

Adj R^2^ = variance explained including age, sex, and BMI

=
